# Talk2Me: Automated linguistic data collection for personal assessment

**DOI:** 10.1371/journal.pone.0212342

**Published:** 2019-03-27

**Authors:** Majid Komeili, Chloé Pou-Prom, Daniyal Liaqat, Kathleen C. Fraser, Maria Yancheva, Frank Rudzicz

**Affiliations:** 1 School of Computer Science, Carleton University, Ottawa, Ontario, Canada; 2 Li Ka Shing Knowledge Institute, Saint Michael’s Hospital, Toronto, Ontario, Canada; 3 Vector Institute for Artificial Intelligence, Toronto, Ontario, Canada; 4 Department of Computer Science, University of Toronto, Toronto, Ontario, Canada; 5 National Research Council Canada, Ottawa, Ontario, Canada; 6 WinterLight Labs, Toronto, Ontario, Canada; 7 Surgical Safety Technologies, Toronto, Ontario, Canada; Newcastle University Institute for Health and Society, UNITED KINGDOM

## Abstract

Language is one the earliest capacities affected by cognitive change. To monitor that change longitudinally, we have developed a web portal for remote linguistic data acquisition, called Talk2Me, consisting of a variety of tasks. In order to facilitate research in different aspects of language, we provide baselines including the relations between different scoring functions within and across tasks. These data can be used to augment studies that require a normative model; for example, we provide baseline classification results in identifying dementia. These data are released publicly along with a comprehensive open-source package for extracting approximately two thousand lexico-syntactic, acoustic, and semantic features. This package can be applied arbitrarily to studies that include linguistic data. To our knowledge, this is the most comprehensive publicly available software for extracting linguistic features. The software includes scoring functions for different tasks.

## Introduction

Between 8% and 10% of the North American population has some speech disorder, including 3 million stutterers, and 7.5 million individuals with dysarthria (caused, e.g, by cerebral palsy, Parkinson’s, or multiple sclerosis) according to the U.S. National Institute of Health. Moreover, since linguistic change is often among the first symptoms of neuro-degenerative cognitive decline, the broader set of speech and language disorders are expected to increase with the rising prevalence of dementia in the aging population [1]. It is therefore imperative to build tools for earlier detection and management of change in language. To the extent to which these tools will be based on machine learning, this will require large datasets; unfortunately, the available data tend to be prohibitively small for rarer diseases, and prohibitively difficult to collect for more at-risk populations. We therefore developed a language assessment tool, called Talk2Me, designed for large-scale self-administered collection of spoken and written language data. This includes new open-source software for feature extraction, a publicly-available data set on which those features were applied, and analysis of relevant linguistic patterns in those features.

After describing the dataset and the feature extraction framework, we present examples of how the dataset might be used in practice, including. This includes unsupervised learning to detect clusters of participants in unlabelled data, and analyzing the relationships between different features within a single task, as a means to reduce redundancy in prospective data collection. We also apply normative healthy control data, of the type we obtain, to the classification of Alzheimer’s disease (AD) in a smaller dataset.

### Assessing AD with automated analysis of language

Language decline is one of the most salient symptoms of AD. Linguistic and acoustic features, such as information content, noun-to-pronoun ratios, and changes to the pitch contour, are indicative of cognitive decline [2–4]. Connected speech is often used for assessing AD, as it provides insight into semantic processing and syntactic complexity [5]. In a study with 30 healthy participants and 36 participants with AD, Meilán *et al* extracted temporal and acoustic features from speech of simple sentences spoken by older adults with and without AD, and achieved an accuracy of 84.8% in a binary classification task (Healthy vs. AD) [6]

Often, connected speech is elicited through the picture description task. Forbes-McKay and Venneri [7] found that deficiencies in semantic processing were apparent not only in participants with AD, but also in those diagnosed with mild or moderate forms of cognitive decline. Jarrold *et al* [8] collected speech from 48 participants completing a picture description task. They then automatically extracted lexical and acoustic features and identified participants with dementia using different machine learning approaches. Similarly, de Lira *et al* extracted linguistic features from transcripts of 121 older adults completing a picture description task and concluded that participants with AD displayed more word-finding difficulties, revisions, and repetitions than healthy controls [9]. In a study involving 48 participants, Giles *et al* [10] used information content to classify participants into four categories of impairment: none, minimal, mild, and moderate. Interestingly, they found that participants with minimal cognitive decline had significant impairments in the production of information units, indicating that decline in the use of semantic content can be detected at early stages of cognitive impairment.

DementiaBank [11] is a popular dataset for studying language in AD in which 167 adults with dementia and 97 adults without, all above the age of 44, completed the ‘Cookie Theft’ picture description task, from the Boston Diagnostic Aphasia Examination [12], which includes the raw audio, the textual transcripts, and the validated clinical assessments. Participants completed these tasks once a year along with a mini-mental state examination. However, longitudinal data points in DementiaBank, especially for AD participants, are sparse. On DementiaBank, Yancheva and Rudzicz [13] automatically detected information content using word embeddings for binary classification of AD and achieved an F-score of 0.74. On the same dataset, Wankerl *et al* employed an *n*-gram based approach and built two language models, one for AD participants and one for healthy participants and achieved a classification accuracy of 77.1% [14]. Fraser *et al* used over 370 automatically extracted acoustic, lexical, and syntactic features to detect AD [2] based on acoustics and speech transcripts.

Other tasks have been used to study the relationship between language and AD. Kirshner *et al* found that people with AD had difficulty on naming tasks even though their language showed no qualitative signs of deterioration, except perhaps that they used more generic terms instead of specific ones [15]. Other work has explored verbal fluency [16] and story recall [3], for example, as means to assess AD.

### Interfaces for self-administered data collection

Clinical cognitive assessments are generally performed in person using physical booklets directly with a clinician. Recently, there has been a push for more remote approaches including telephone-based versions of existing cognitive assessments such as the MMSE and the Montréal Cognitive Assessment (MoCA) [17]. Rapcan *et al* [18] administered a battery of language assessments over the telephone and found that speech features (e.g., number of pauses, length of utterances) could be reliably extracted by telephone recordings and did not significantly differ from in-person clinic recordings. Using an interactive-voice-response telephone system, Yu *et al* [19] extracted speech features and achieved an AUC of 0.77 in classifying between healthy and cognitively impaired participants.

Van Mierlo *et al* [20] built a web- and telephone-based system for administering cognitive self-tests as a method of automatic screening. In their study, 117 participants used their system and were classified into one of the following categories: subjective cognitive decline, mild cognitive impairment, and dementia. They achieved an AUC of 0.86 with the web-based system, and an AUC of 0.78 on the telephone assessment. The tasks employed in that work, however, did not include free-form speech or language production, which is our focus here.

## Materials and methods

Talk2Me collects data through tasks similar to those used in standard assessments of cognition (including the Mini-Mental State Examination [21], the Montréal Cognitive Assessment [22], and the Western Aphasia Battery [23]). Users register on the website and provide consent, then complete a demographics survey (S1 Fig). The survey collects information on their sex, age, ethnicity, language fluency, education level, country of origin, and country of residence. Users are also asked if they have ever been diagnosed with dementia, if they are currently taking dementia medication, and if they have been a regular smoker of tobacco within the last 3 years. After answering the survey questions, they can then complete multiple sessions of data collection, some through typing and others through speaking. In order to be as generic as possible, no restrictions are placed on the environment or channel, except that the browser must support HTML5, which is the case for all major browsers. The source code for this tool is being made available publicly (https://github.com/SPOClab-ca/talk2me_interface).

All data were recorded given informed consent by the participants, according to Research Ethics Board protocol #31127 of the University of Toronto, which specifically approved this study.

### Language tasks

Website users complete eight different types of language task, each designed to evaluate various aspects of cognition. During each session, users complete one or more instances of each task, with each instance corresponding to a different stimulus (e.g., a different word to define, or different picture to describe), as summarized in Table 1.

**Image naming** In each image naming session, six pictures are displayed and the participant types the name of each object depicted. Images are taken from the Caltech-256 Object Category dataset [24].
**Picture description** Participants verbally describe images that convey more complex scenes that show interacting objects. There is no time constraint on this task, although participants are encouraged to speak for at least one minute. Images used for this task include the *‘Cookie Theft’* picture from the Boston Diagnostic Aphasia Examination [12], the *‘Picnic scene’* from the Western Aphasia Battery [23], twelve re-distributable images from Flickr (https://www.flickr.com/), and twenty-nine images from the Webber Photo Cards: Story Starters collection [25].
**Fluency** In each session, participants type as many words as possible that match the category. Categories typically consist of a semantic variant (e.g., types of animal) or a phonemic variant (e.g., words that begin with *F*). Verbal performance on this task can differentiate a variety of conditions, including traumatic brain injury [26] and dementia [27].
**Story recall** A short story is displayed to the participant. The text then disappears, as expected, and participants verbally re-tell the story in their own words. There is no time constraint on either phase, but participants are encouraged to speak for at least a minute. Stories used in this task are the *‘My Grandfather’* short story [28], the *‘Rainbow’* passage [29], and the *‘Limpy’* passage (http://itcdland.csumb.edu/~mimeyer/CST251/readingpassages.html) which are standardized among speech-language pathologists to assess speaking and memory skills.
**Vocabulary** Participants define five words by typing definitions using their own words. Words used in this task are taken from the Brown corpus [30]. Each word is assigned a difficulty based on its age-of-acquisition, derived from the Kuperman norms [31]. Specifically, the set of all words is sorted by increasing age-of-acquisition and subsequently trisected into partitions of equal size, uniformly across scores, representing ‘easy’, ‘moderate’, and ‘difficult’ words.
**Winograd schema** The Winograd Schema challenge [32] consists of questions with two possible answers (e.g., one stimuli is ‘*The trophy could not fit into the suitcase because it was too big. What was too big—the trophy or the suitcase?*’). Instances are taken from the publicly available Winograd Schema challenge (https://www.cs.nyu.edu/davise/papers/WS.html). Participants simply select an answer from the available pair.
**Word-colour Stroop** In the Stroop inference task [33], the user is presented with the name of a colour, presented in a coloured typeface. The user says the colour of the given font out loud, ignoring the orthography. The Stroop test has a high degree of discriminative power in Alzheimer’s disease [34], depression [35], and bipolar disorder [36], for example.
**Self-reported disposition** Participants answer five questions taken from a validated short-form version [37] of the Geriatric Depression Scale (GDS), which is a 30-item self-assessment used to identify depression in the elderly [38]. We collect these responses, since mood can affect a person’s performance in language tasks, and since a focus on dementia is ongoing in a parallel study. From the GDS, we ask yes/no questions on life satisfaction, general happiness, and everyday activities. Participants are also asked to rate their current mood on a scale from 1 (very sad) to 10 (very happy).

**Table 1:** Modality of language tasks, and the number of unique stimuli per session for each task.

Task	Mode	# of stimuli
Image naming	Text	6
Picture description	Audio	1
Fluency	Text	1
Story recall	Audio	1
Vocabulary	Text	6
Winograd schemas	Multiple-choice	5
Word-colour Stroop	Audio	18
General disposition	Multiple-choice, Likert scale	5

## Talk2Me database

In this section, we describe the demographics of individuals in the database, then we describe how different tasks are scored. Lastly, we describe the extracted features. A task *score* is a quantitative measure of how well that task was performed towards some *goal*. Naturally, this only applies to tasks that have an explicit purpose to be achieved. Unlike scores, *features* don’t directly measure success in performing a task, but rather evaluate intrinsic aspects of how the task was performed.

### Demographics

**Fig 1:**
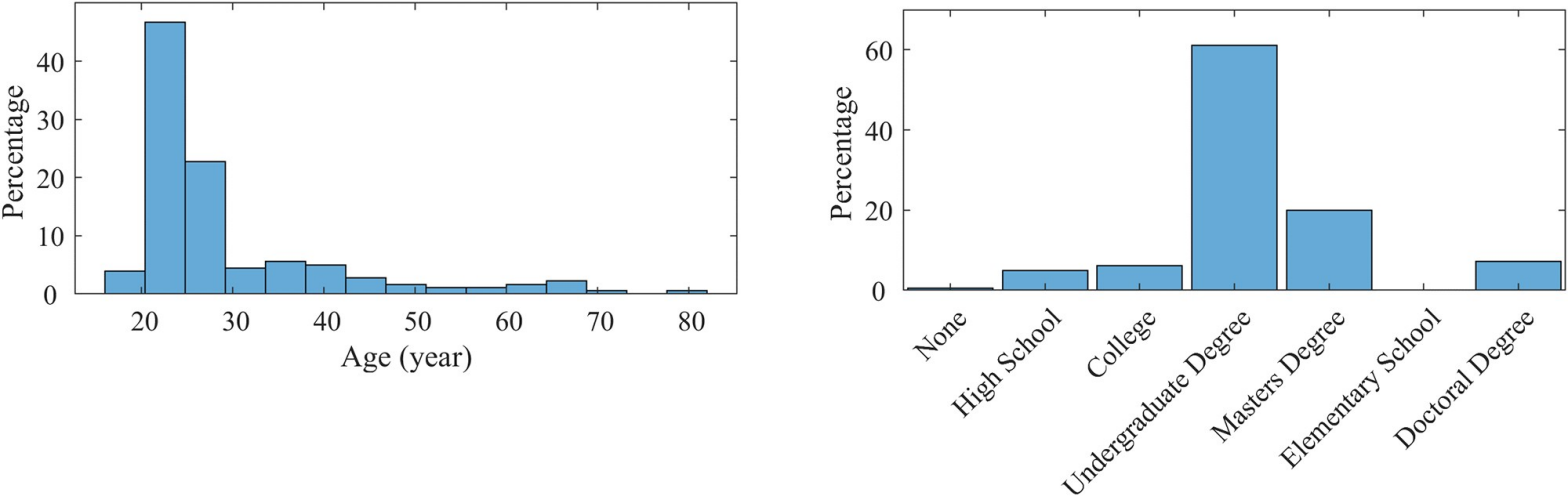
Demographics: Age and education distribution of participants.

Collection of this database is ongoing, and subsequent releases or “snapshots” that we make publicly available will be versioned and time-stamped. We report results and analysis on 1369 sessions completed by 339 unique users, of whom 206 have completed more than one session. The released dataset includes sessions from all participants who have agreed to the public release of their data, and contains 1033 sessions from 196 users. Participants were recruited on a voluntary basis, self-assessing for an adequate level of proficiency in English. 96% of users report being native or fluent speakers of English and 92% of users report being Canadian residents but the tool is not built for any particular country or accent; 3% are from United States and the rest are from other countries. We do not restrict age, sex, or other demographics. While most participants using Talk2Me are less than 30 years of age, approximately 50 users are older adults and 36% are female. Fig 1 shows the distribution of age and education level over all participants.

### Scoring the tasks

We automatically transcribe audio files for the picture description, story recall, and word-colour Stroop tasks with Kaldi, an open-source speech recognition toolkit [39], using a long short-term memory neural network with i-Vector input [40] and a reverberation model, trained on the Fisher data [41]. An *ad hoc* evaluation of a random 10% of transcripts generated from the story recall task reveals a word-error rate of 28.08%, which is approximately state-of-the-art for large-vocabulary speech recognition [42]. Each transcript is then aligned with its corresponding audio file using the Gentle forced aligner (https://github.com/lowerquality/gentle), and then segmented into sentences based on pitch, pause, and parts-of-speech features [43]. The fully segmented transcripts are then scored, as described below.

#### Image naming scores

Stimuli used in the image naming task are taken from the Caltech-256 Object Category dataset [24], which are labeled. We measure the similarity (on [0..1]) between user input and the set of provided annotations using Wu-Palmer Similarity (WuP) [44] on the ontology provided by WordNet [45]. WordNet is a lexical database that groups English words into synonym sets, and maintains a number of relations among these sets and their members. WuP returns a score denoting how similar two synonym sets (*c*_1_ and *c*_2_) are, based on the depth of the two senses in the ontological graph, from the root node, and that of their least common subsumer *LCS* (i.e., their most specific ancestor node). Specifically,

$$\begin{array}{c} sim_{wup}=\frac{2\times depth(LCS\left(c_{1},c_{2}\right)}{depth\left(c_{1}\right)+depth\left(c_{2}\right)}. \end{array}$$ (1)

**Fig 2:**
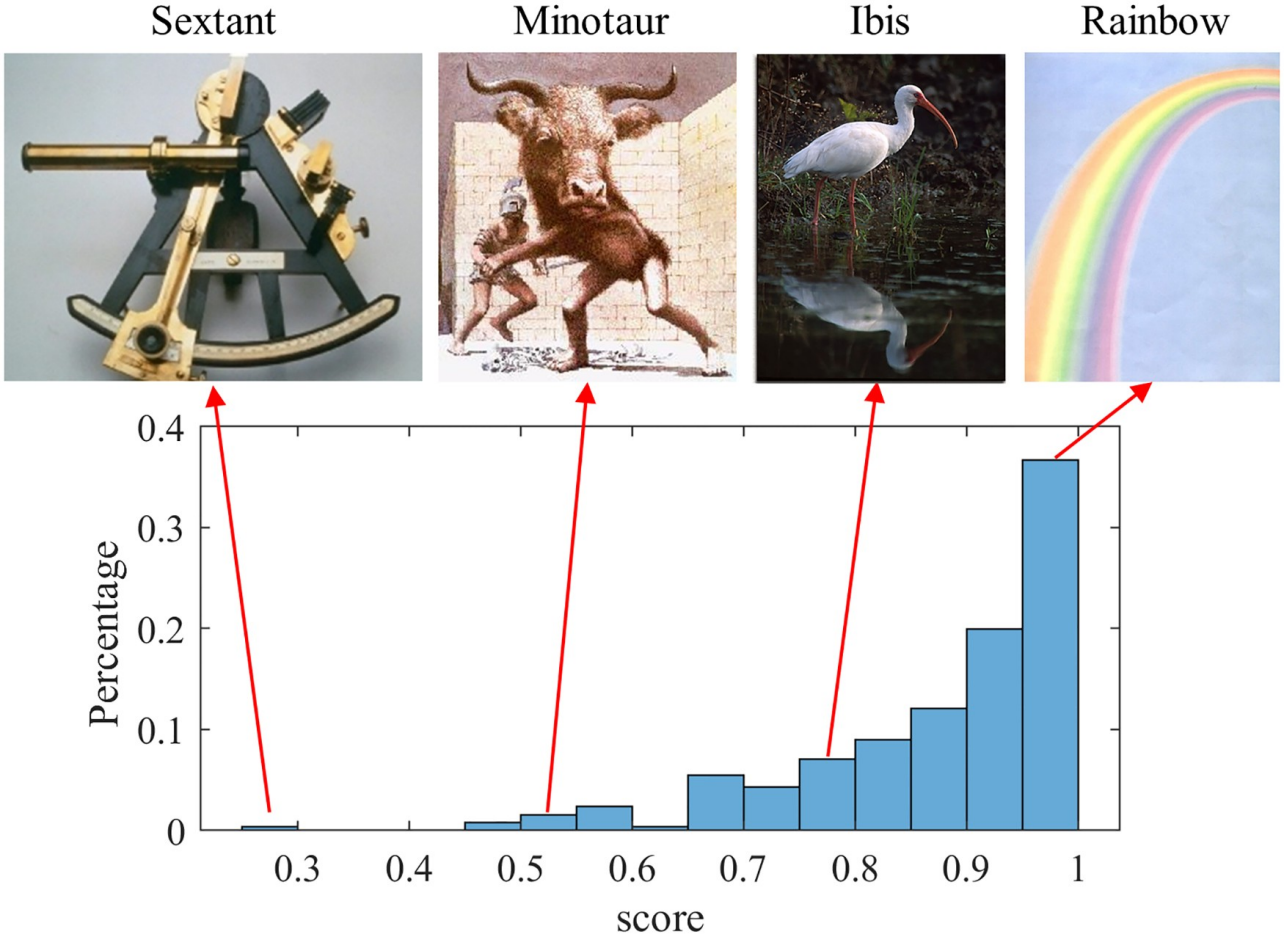
Image naming task: Distribution of average scores for different images. We provide examples of images that appear simple and challenging.

Since words can have multiple senses, we choose the most frequent one. There are 257 stimuli in this task. The average score per stimulus is computed and the distribution of the average score for each stimulus is summarized in Fig 2. The overall average score for this task is 0.89 with a variance of 0.02 and a skewness of −1.5.

#### Picture description scores

Picture description is often scored in terms of both syntactic and semantic properties, such as agrammatical deletions and ‘emptiness’, respectively [12]. For the former, we measure language complexity automatically using Lu’s Syntactic Complexity Analyzer (SCA) [46]. For the latter, we count the number of information content units (ICUs) in produced transcripts. These ICUs constitute entities, actions, or relations in the scene, and were initially determined through annotation by speech-language pathologists. Since participants may describe an ICU in different terms (e.g., ‘mom’ instead of ‘mother’, or ‘kid’ instead of ‘boy’), we use the Lin Similarity (LS) metric [47] from NLTK to account for lexical variety. LS computes the similarity of two synonym sets (*c*_1_ and *c*_2_) based on the Information Content (IC) of the the Least Common Subsumer and that of the two input synonym sets. Specifically,

$$\begin{array}{c} sim_{LS}=\frac{2\times IC(LCS(c_{1},c_{2}))}{IC\left(c_{1}\right)+IC\left(c_{2}\right)}. \end{array}$$ (2)

For the words in the input sentence, all possible senses are considered and we accept an input word as an ICU if the similarity of its closest synonym set is greater than 0.75, determined empirically. For each picture, 10 examples were randomly selected and manually verified against different thresholds. If a word is determined to be synonym of an ICU in the context of the picture but their similarity does not satisfy the above threshold, the word is manually added to the list of ICUs for that picture. A very low value for the threshold results in many words being falsely detected as ICUs. A very large value results in many ICUs not be detected and therefore many synonyms should be added manually to the list of ICUs. The threshold of 0.75 empirically balanced accurately detecting ICUs while minimizing manual annotation.

**Fig 3:**
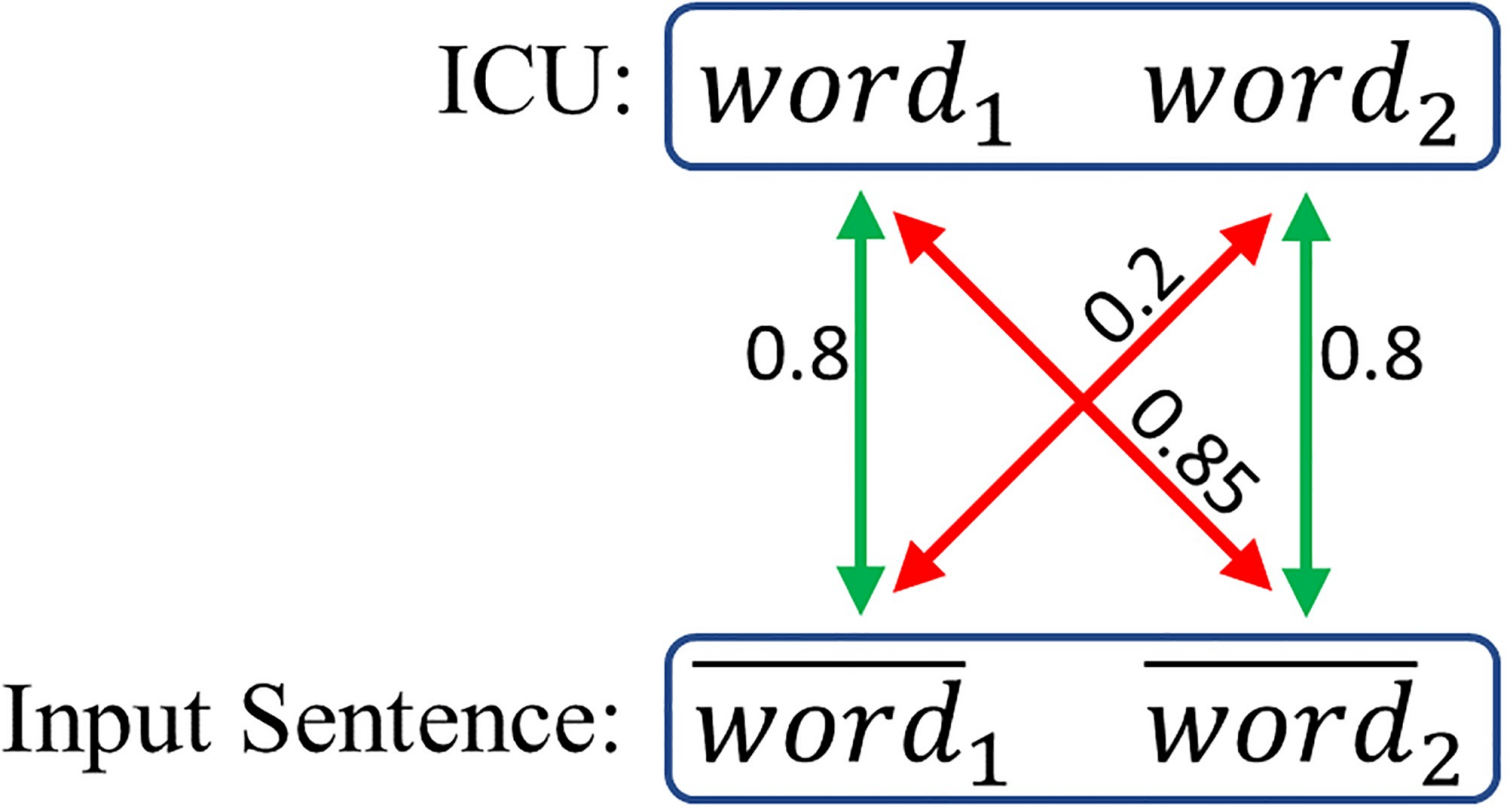
Picture description task: Optimal pairing is found by solving a maximum weight matching in bipartite graphs. Using a threshold of 0.75, a greedy algorithm would result in pairing *word*_1_ with ${\bar{word}}_{2}$. The optimal pairing of *word*_1_ with ${\bar{word}}_{1}$ and *word*_2_ with ${\bar{word}}_{2}$ is obtained with maximum weight matching.

ICUs can also take the form of multi-word phrases (e.g., ‘*hard drive*’). To compare an ICU with *m* words with an input window of *n* words (where *m* ≥ *n* by definition), each word in the ICU must be paired with a word in the input. Note that, as illustrated in Fig 3, a greedy strategy can result in suboptimal pairings, called maximum weight matching in bipartite graph theory. Therefore, using maximum weight matching [48], an ICU is detected if the similarities for all words in the candidate are greater than the empirical threshold 0.75. Note that this does not incorporate grammatical dependencies or negations.

**Fig 4:**
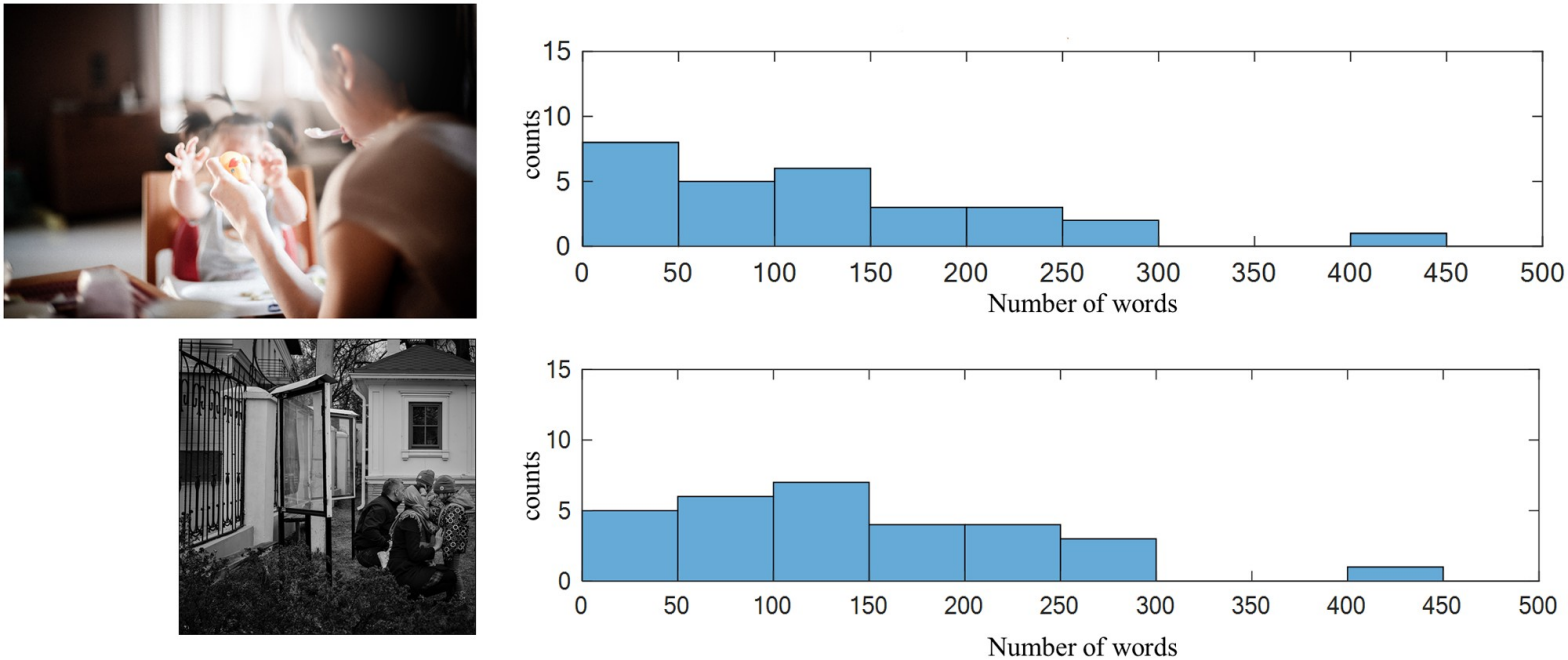
The picture description task: Distributions of the number of words for two of the picture stimuli (shown) with longer descriptions. The top image is a redistributable proxy of image 17 from the Webber collection.

**Fig 5:**
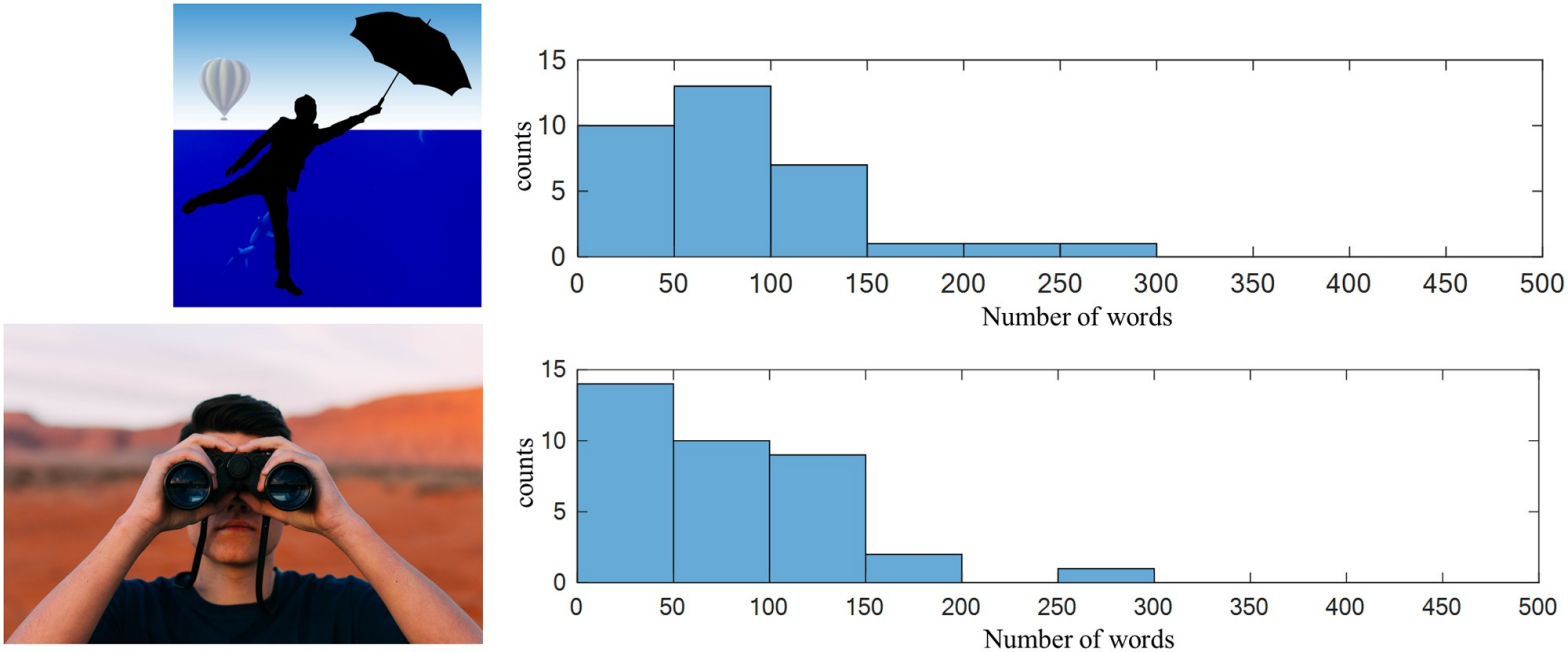
The picture description task: Distributions of the number of words for two of the picture stimuli (shown) with shorter descriptions. The top image and bottom images are redistributable proxies of images 41 and 42, respectively, from the Webber collection.

Pictures can have a relatively arbitrary number of ICUs. Some pictures elicit more or less speech, as shown in Figs 4 and 5, respectively.

#### Fluency scores

We extract the same scores as in the Wisconsin Longitudinal Study [49] for the same type of task, including the number of tokens in a category and the number of tokens out-of-category. For the semantic fluency task, we manually construct dictionaries for each stimulus, based on a subset of user responses (e.g., the *‘animal’* dictionary contains the words *‘lion’*, *‘tiger’*, and *‘cat’*. To determine if a word is in- or out-of-category, we first check if it belongs to any of the dictionaries. If the word is not found, we use WordNet to check if the category word is its hypernym. For the letter fluency, we check that each word begins with the given letter, and then verify that the word exists by checking if it can be found in WordNet. Alternative dictionaries may be used, in general.

**Fig 6:**
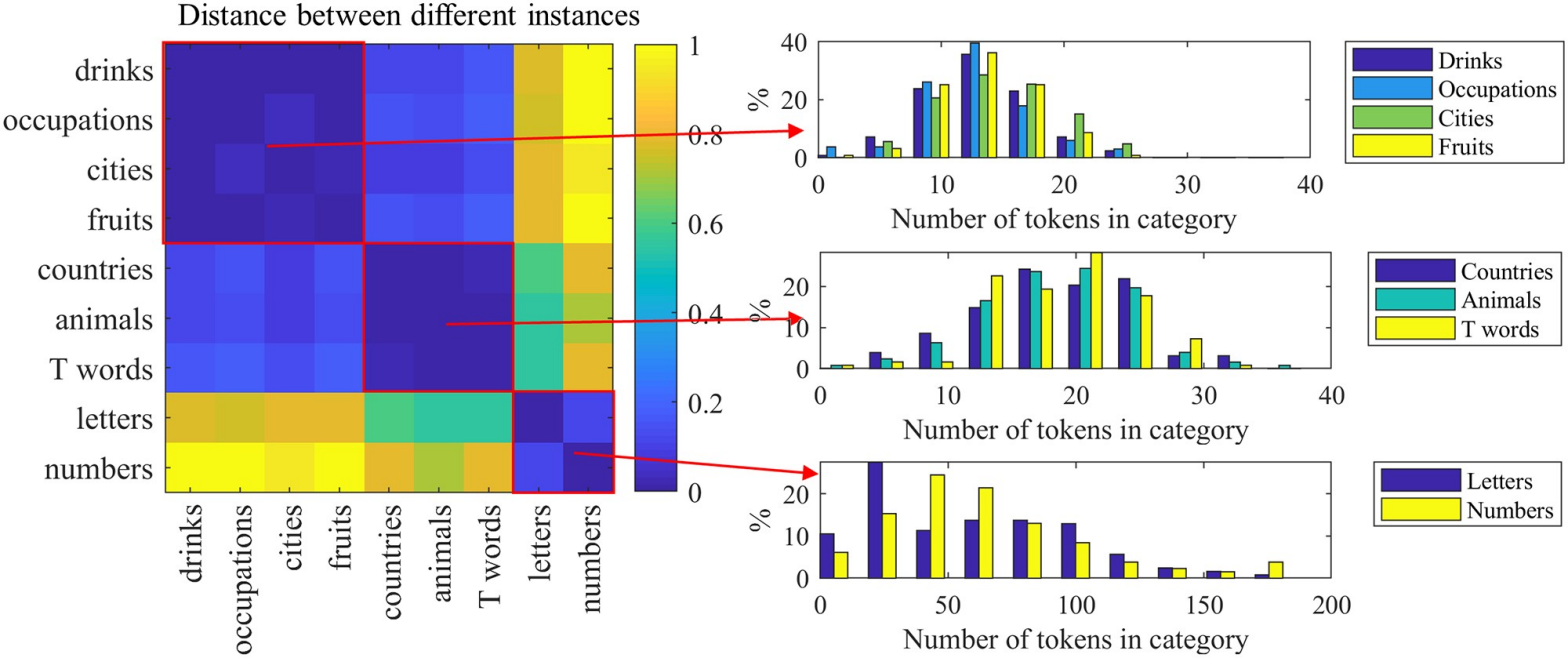
Fluency task: KL distances between distributions of the number of the tokens *in* category for different instances of the fluency task. The actual distributions are shown on the right.

**Fig 7:**
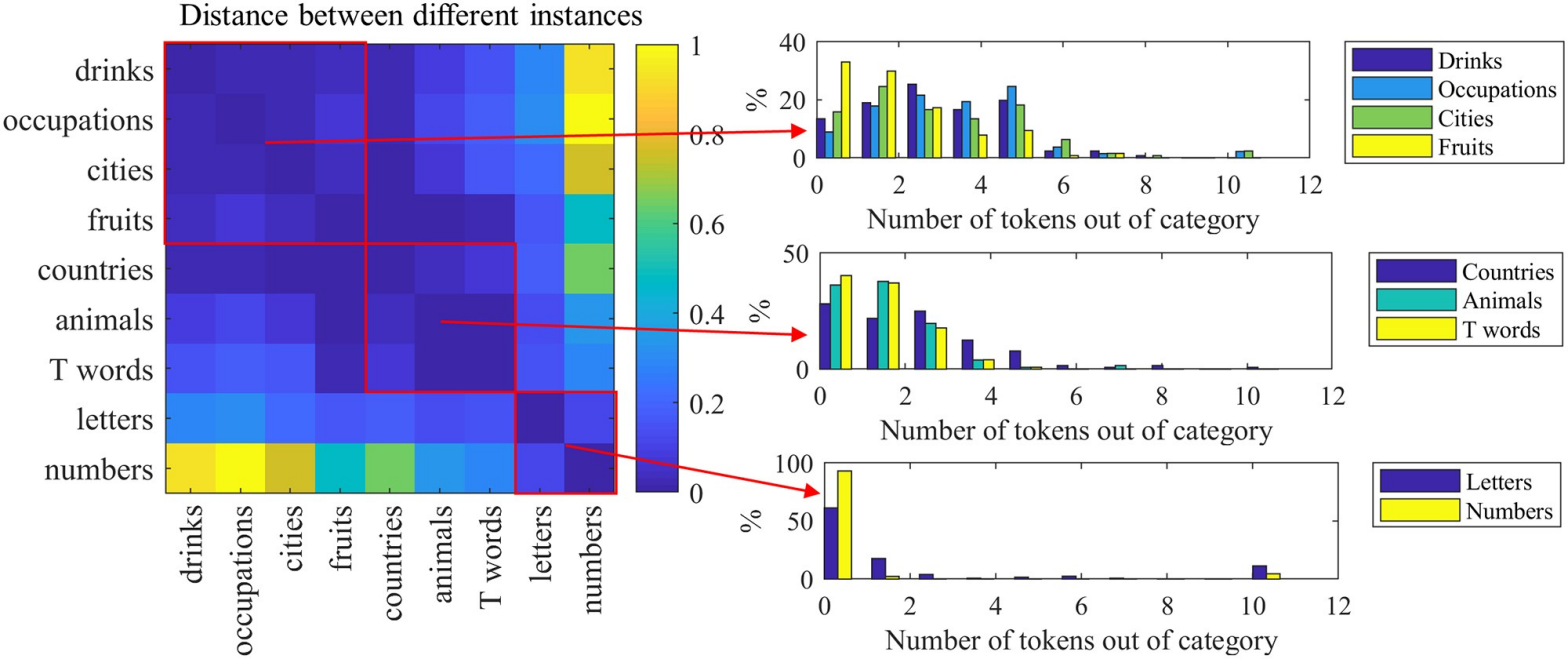
Fluency task: KL distances between distributions of the number of the tokens *out* of category for different instances of the fluency task. The actual distributions are shown on the right.

Alzheimer’s disease, for example, has a greater impact on semantic fluency than on other types of fluency [16, 50]. Fig 6 shows the pairwise Kullback—Leibler (KL) divergence between the distributions of the number of tokens in-category, for different instances of the fluency task. For visualization, values are linearly normalized on [0..1]. Dark blue elements indicate smaller distances between distributions. Here, *‘drinks’*, *‘occupations’*, *‘cities’*, and *‘fruits’* show similar distributions. As one may expect, *‘letters’* and *‘numbers’* have also similar distributions. The distributions of the number of tokens *out* of category for different instances are examined in Fig 7.

These results suggest that latent subgroups exist in the fluency task, which may be useful in mitigating the practice effect that often occurs in longitudinal analysis [51].

#### Story recall scores

**Fig 8:**
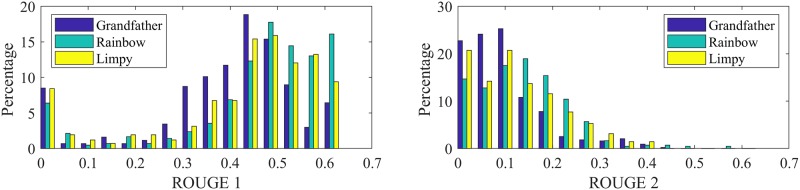
Story recall: Distribution of the average ROUGE-1 (ROUGE-2) scores for different stories on right (left).

We transcribe the audio recordings of story recall, and score the task using the ROUGE score (i.e., ‘recall-oriented understudy for gisting evaluation’) [52]. ROUGE is typically used to evaluate automatic summarization software, and compares a candidate summary to a list of reference summaries using the overlap of their *n*-grams. When scoring the story recall task, we use the original text of the short story as the reference and the transcript of the participant’s story retelling as the candidate. We extract ROUGE metrics on unigrams (ROUGE-1) and on bigrams (ROUGE-2). Fig 8 shows the distribution of these scores for different stories, including the ‘Grandfather’ passage, whose lower scores suggest that it is harder to recall.

#### Vocabulary scores

We use the BLEU measure (i.e., ‘bilingual evaluation understudy’) [53] to score the vocabulary task. BLEU is similar to ROUGE in that it compares oracle-provided reference sentences and candidate sentences, but its focus is precision rather than recall. Specifically, given a brevity penalty:

$$BP=\{\begin{array}{ll} 1 & \text{if}\;r<c\\ e^{1-r/c} & \text{if}\;r\geq c \end{array}$$ (3)

where *c* is the number of word tokens in the candidate and *r* is the nearest length among references, and

$$\begin{array}{c} p_{n}=C/N \end{array}$$ (4)

is the *n*-gram precision given the number *C* of *n*-grams in the candidate that are in at least one reference and the total number *N* of words in the candidate, then:

$$\begin{array}{c} BLEU=BP{\left(p_{1}p_{2}...p_{n}\right)}^{1/n} \end{array}$$ (5)

**Fig 9:**
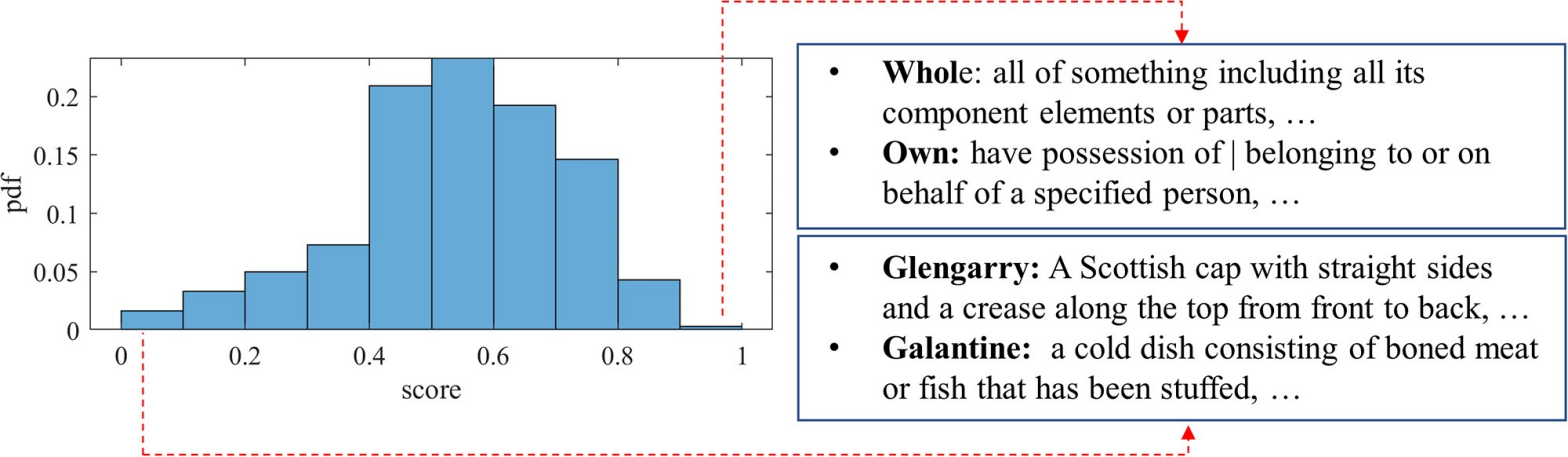
Vocabulary task: Distribution of the average BLEU score of different stimuli. Some examples with very high and low scores are also provided on the right, from references.

In our case, the user provides the candidate definition, and reference definitions are derived from WordNet [54], Wiktionary (http://www.igrec.ca/projects/wiktionary-text-parser/, and the Merriam-Webster dictionary (https://www.dictionaryapi.com/). There are 301 different stimuli. The average BLEU score per stimulus is computed and the distribution of the average score of the stimuli is shown in Fig 9. We provide examples of vocabulary items with very high and very low scores. The average score of the Vocabulary task is 0.49 with variance of 0.03 and skewness of -0.41. Stimuli around the average may be good candidates for future studies of vocabulary.

#### Winograd schema scores

**Fig 10:**
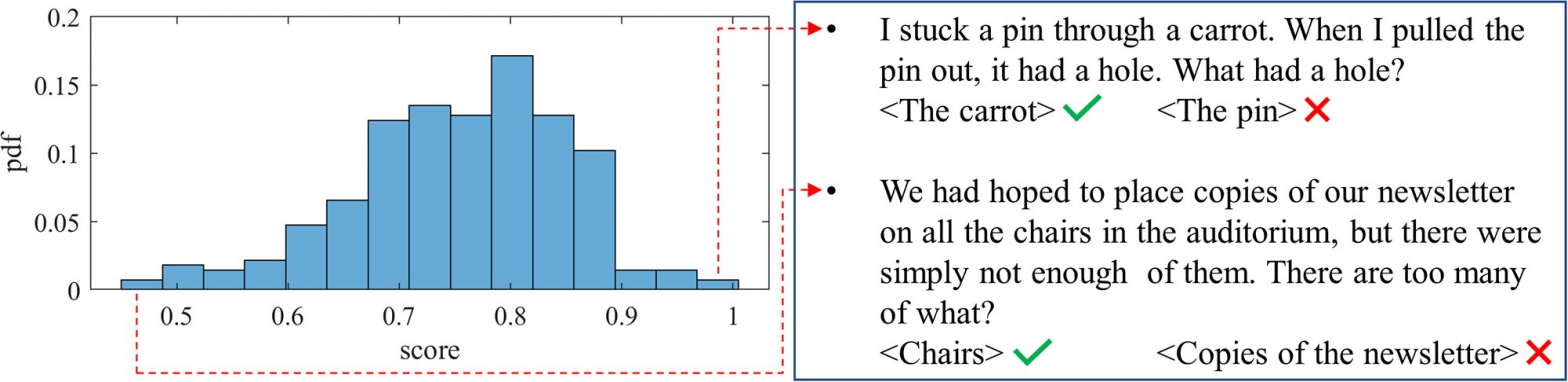
Winograd task: Distribution of the average score given different stimuli. Some examples with very high and very low scores are provided on the right.

The dataset used for the Winograd schema is annotated with correct answers. Participants receive a score of 1 for every correct response, and 0 otherwise. There are 274 Winograd stimuli. The average score per stimulus is computed and the distribution of the average score of the stimuli is shown in Fig 10. The average score of the Winograd task is 0.75 with a variance of 0.01 and a skewness of −0.46. Similar to the vocabulary task, the stimuli around the average may be a good candidate for future studies.

### Feature extraction

We extract both textual features where available (including transcripts from speech recognition), and acoustic features from audio, as described below.

From text and transcribed audio of the image naming, fluency, story recall, and vocabulary tasks, we extract lexical, syntactic, semantic, and pragmatic features, as described in Table 2.

**Table 2:** Extracted lexical, syntactic, semantic, and pragmatic features for image naming (IN), picture description (PD), fluency (F), story recall (SR), and vocabulary (V) tasks. We do not extract any text features for the Winograd schema or the word-colour Stroop task.

		IN	PD	F	SR	V
**Lexical**	Word	✔	✔	✔	✔	
	Fillers		✔	✔	✔	✔
	Vocabulary richness		✔	✔	✔	✔
	Readability		✔	✔	✔	✔
	Polarity		✔	✔	✔	✔
	POS counts & ratios		✔	✔	✔	✔
	Lexical norms	✔	✔	✔	✔	✔
**Pragmatic**	LDA		✔		✔	
	RST		✔		✔	
**Semantic**	WordNet		✔	✔	✔	✔
	Cosine measures		✔	✔	✔	✔
**Syntactic**	Lu		✔		✔	✔
	Parse trees		✔		✔	✔
	Density	✔	✔	✔	✔	✔

#### Lexical features

We automatically extract features related to each word (e.g., the number of syllables per word, and the number of characters per word). We count the number of fillers (e.g., *“uh”, “um”*) and normalize by the total number of word tokens in the sample.

To compute vocabulary richness, we calculate the type-token-ratio and the moving-average-type-token-ratio [55] with window sizes of 10, 20, 30, 40, and 50. We also calculate the Brunet index (Eq 6) and the Honoré statistic (Eq 7) [56]; i.e.,

$$\begin{array}{c} BI=N^{\left(U^{-0.165}\right)}, \end{array}$$ (6)

where *N* is total number of word tokens and *U* is the total number of unique word types, and

$$\begin{array}{c} HS=\frac{100\log N}{1-\frac{N_{1}}{U}}, \end{array}$$ (7)

where *N* is the total number of word tokens, *U* is the total number of unique word types, and *N*_1_ is the number of *hapax legomena* (i.e., words used only once).

The readability of transcripts is calculated by the Flesch reading score (Eq 8), and the Flesch-Kincaid grade level (Eq 9) [57]; i.e.,

$$\begin{array}{c} F=206.835-1.015\frac{total\;words}{total\;sentences}-84.6\frac{total\;syllables}{total\;words} \end{array}$$ (8)

and

$$\begin{array}{c} FK=0.39\frac{total\;words}{total\;sentences}+11.8\frac{total\;syllables}{total\;words}+15.59 \end{array}$$ (9)

We measure the polarity of transcripts by computing averages and standard deviations of norms derived from the Multi-Perspective Question Answering (MPQA) lexicon [58] and the Stanford Sentiment analyzer [59]. The MPQA lexicon provides values of polarity of words as *“strong negative”, “strong positive”, “weak positive”*, or *“weak negative”*. The Stanford Sentiment analyzer provides values of polarity of words as *“very negative”, “very positive”, “neutral”, “negative”*, or *“positive”*.

We extract mean values of frequency, age-of-acquisition, imageability, familiarity, arousal, dominance, and valence based on lexical norms. We compute the mean frequency (with which a word occurs in a corpus) of words based on the SUBTL frequency norms [60]. Age-of-acquisition (i.e., the age at which a person learned a word), imageability (i.e., the ease at which a word can give rise to a mental image), and familiarity (i.e., how often a word is used, seen or heard) are determined from the Bristol [61] and Gilhoolie-Logie ratings [62]. Arousal (i.e., the intensity of emotion), dominance (i.e., the degree of control), and valence (i.e., the pleasantness) of words are derived from the Affective Norms for English Words (ANEW) ratings [63] and the Warriner norms [64]. We also obtain average values for psycholinguistic measures from the Linguistic Inquiry and Word Count (LIWC) corpus [65] and the Receptiviti platform (https://www.receptiviti.ai/liwc-api-get-started).

#### Syntactic features

We count constructs extracted from Lu’s Syntactic Complexity Analyzer (SCA) [46]. SCA computes various ratios involving T-units (i.e., main clauses plus their dependent clauses) and complex nominals (i.e., groups of words that describe an entity). We compute the Yngve measure [66], which is computed from Stanford context-free parse trees and quantifies to what extent a sentence is left-branching rather than right-branching. We extract propositional [67] and content density [3], respectively:

$$\begin{array}{c} density_{prop}=\frac{verbs+adjectives+adverbs+prepositions+conjunctions}{words}, \end{array}$$ (10)

and

$$\begin{array}{c} density_{content}=\frac{nouns+verbs+adjectives+adverbs}{words}. \end{array}$$ (11)

Next, we measure the part-of-speech (POS) counts using the Stanford POS tagger (https://nlp.stanford.edu/software/tagger.shtml). These include adjectives, adverbs, coordinate conjunctions, demonstratives, determiners, function words, inflected verbs, light verbs, nouns, prepositions, pronouns, subordinate conjunctions, verbs. We also compute the following POS ratios:

$$\begin{array}{c} \begin{array}{cc} noun-verb\;ratio & =\frac{\#nouns}{\#verbs}\\ noun\;ratio & =\frac{\#nouns}{\left(\#nouns+\#verbs\right)}\\ pronoun\;ratio & =\frac{\#pronouns}{\left(\#pronouns+\#nouns\right)}\\ subordinate-coordinate\;ratio & =\frac{\#subordinate\;conjunctions}{\#coordinate\;conjunctions} \end{array} \end{array}$$ (12)

#### Semantic features

We compute semantic similarity using the average and minimum cosine distance between each pair of one-hot embeddings of utterances, and the cosine cutoff (i.e., the number of pairs of utterances whose the cosine distance is below a certain threshold). We compute word specificity and ambiguity based on tree depth and the number of senses in WordNet [54]. We also extract multiple WordNet measures of similarity: Resnik [68], Jiang-Coranth [69], Lin [47], Leacock-Chodorow [70], and Wu-Palmer [71].

#### Pragmatic features

We train a general 100-topic latent Dirichlet allocation (LDA) model [72] on the Wikipedia corpus for generalizability. LDA is a generative statistical model used to determine unlabeled topics in a document. For each transcript, we extract the probabilities of each LDA topic. Next, we extract features related to rhetorical structure theory (RST), which is a classic framework for discourse parsing in which partitions of text are arranged in a tree structure by pragmatic relations such as *Elaboration* or *Contrast* [73].

#### Acoustic features

We extract acoustic features from all tasks in which the response is spoken, i.e., the picture description, story recall, and word-colour Stroop tasks. We extract acoustic features with the openSMILE open-source tool [74], which includes features related to formants, loudness, approximations of pitch, including zero-crossing rate and Mel-frequency cepstral coefficients (MFCCs) among others. Additionally, we extract the following features that are not extracted by openSMILE: 1) total duration, 2) total duration of active speech divided by total duration of the sample, 3) mean length of all pauses (pause > 150 ms), short pauses (150 ms < pause < 400 ms), and long pauses (pause > 400 ms), and 4) ratio of pauses > 150 ms to non-silent segments.

## Results

### Correlation across different tasks

**Fig 11:**
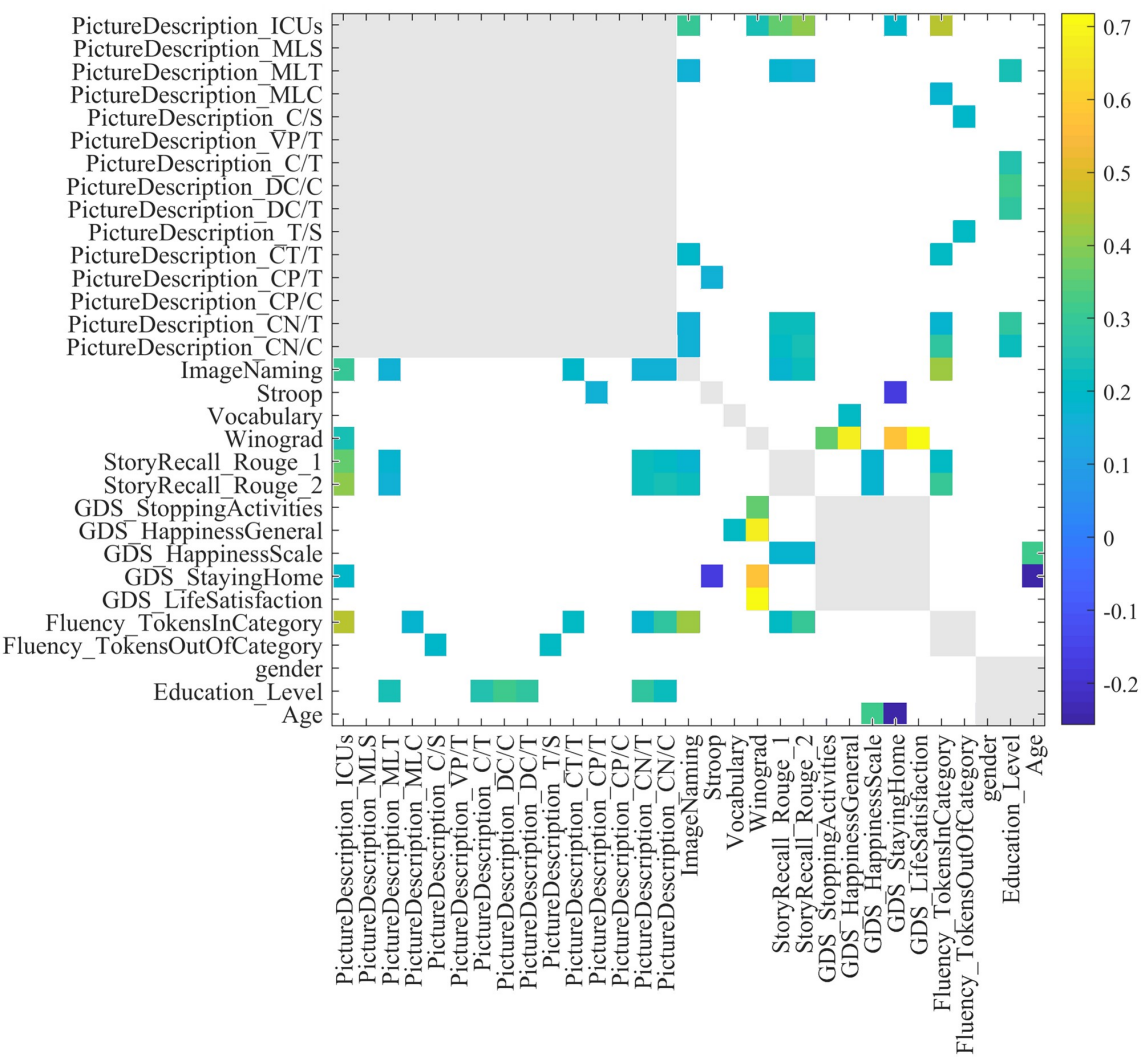
Correlation between scores of different tasks. For a better visualization, same-task correlation values are shown in gray and those that are not significant with respect to *p* = 0.05 are in white. S: # of sentences, T: # of T-units, C: # of clauses, VP: # of verb phrases, CT: # of complex T-units, CN: # of complex nominals, CP: # of coordinate phrases, DC: # of dependent clauses, MLS: Mean length of sentence, MLTU: Mean length of T-unit, MLC: Mean length of clause.

**Fig 12:**
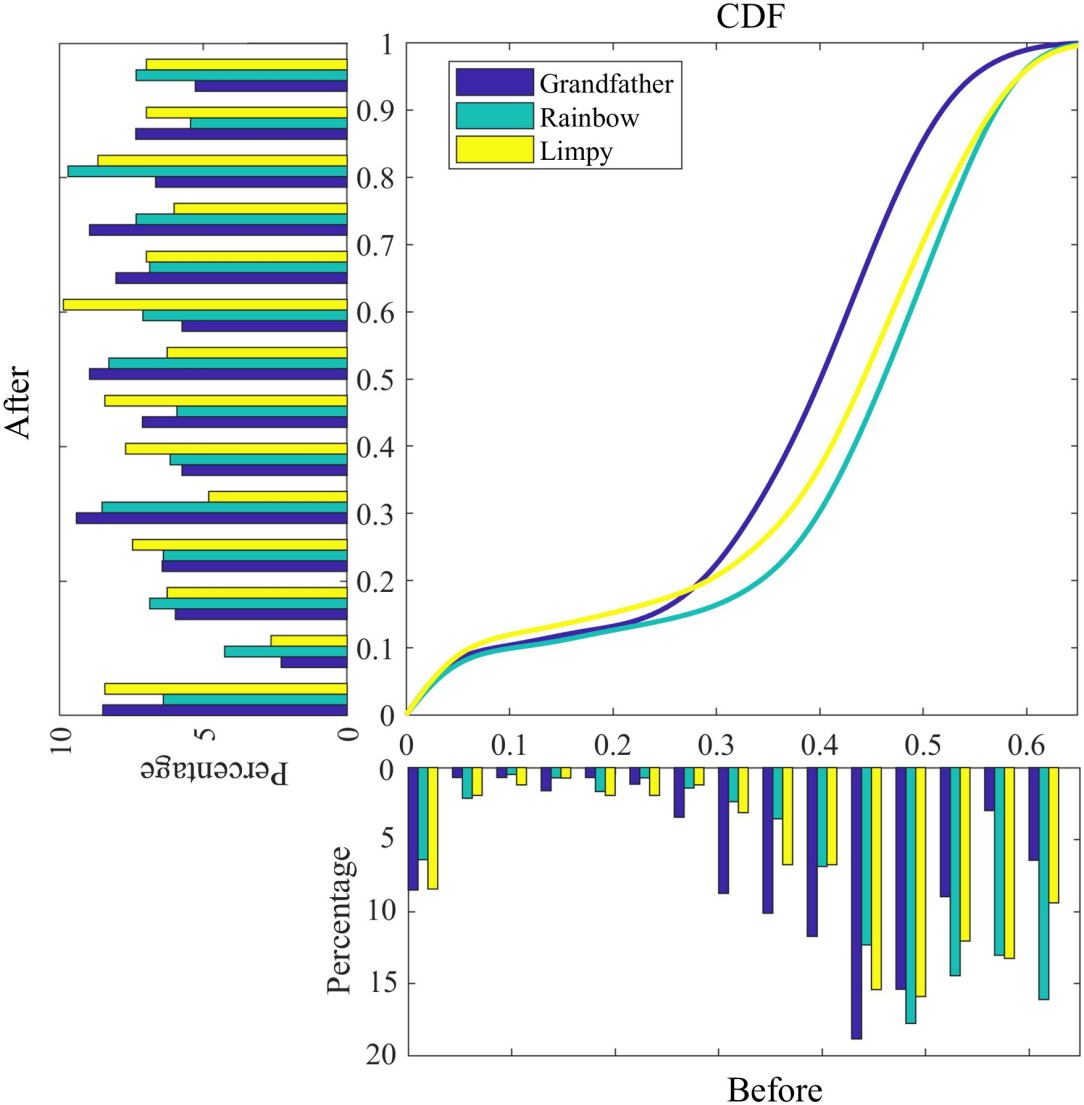
An illustration of score normalization based on a cumulative distribution function, for the story recall task. Bottom: distribution of the original scores (ROUGE-1); Center: cumulative distribution functions; Left: distribution of the normalized scores.

In this section, we evaluate the relations between the performance of subjects on different tasks through correlation analysis. For tasks that are scored with multiple measures, e.g., ROUGE-1 and ROUGE-2 in story recall, we consider all the measures and the results are shown in Fig 11. We also include age, sex, and the education level in the analysis. Scores within the same task are usually very highly correlated across subjects, as one might expect; therefore, for visualization, we only show correlations between scores *across different* tasks. Additionally, correlation values that are *not significant*, with respect to the *p* = 0.05, are also ignored. We have normalized the scores as follows:

Tasks such as picture description have different stimuli, which may affect the scores. To alleviate this effect, scores are equalized according to their cumulative distribution function (CDF). The resulting scores are therefore uniformly distributed between 0 and 1. This technique is also known as ‘histogram equalization’ or ‘dynamic range expansion’ [75]. Fig 12 illustrates this process for story recall. The original scores for the ‘grandfather story’ are lower than the ‘rainbow story’, suggesting that it is a harder story to recall (Fig 12, bottom). This has been alleviated in the normalized scores, where all stories have similar distribution (Fig 12, left). For tasks that involve binary questions, such as Winograd, CDF is not helpful because the probability distribution function is a Bernoulli process. However, in those tasks, there are multiple stimuli per session that allows for computing an average over stimuli. Taking into account the fact that some stimuli are harder than others, we adopt a weighted average strategy such that the effect of ‘hard’ questions are reduced. That is, the average score is more degraded if a subject answers an easy question incorrectly. The weight of a question represents its ‘simplicity’ and is defined as the rate of correct responses to that question, computed over all available responses to that question. For subjects with more than one session, normalized scores are averaged over all available sessions.

In the Fluency task, the number of tokens ‘in category’ has a moderate correlation with the number of ICUs in the picture description task (*ρ* = 0.44, *p* = 9 × 10^−9^) and with performance on the image naming task (*ρ* = 0.43, *p* = 6 × 10^−8^). Similarly, the Rouge-2 score in story recall is correlated with number of ICUs in the picture description task (*ρ* = 0.41, *p* = 2 × 10^−7^). The Winograd task is correlated with the life satisfaction response in the GDS task (*ρ* = 0.71, *p* = 5 × 10^−20^).

### Principal component analysis of scores within tasks

**Fig 13:**
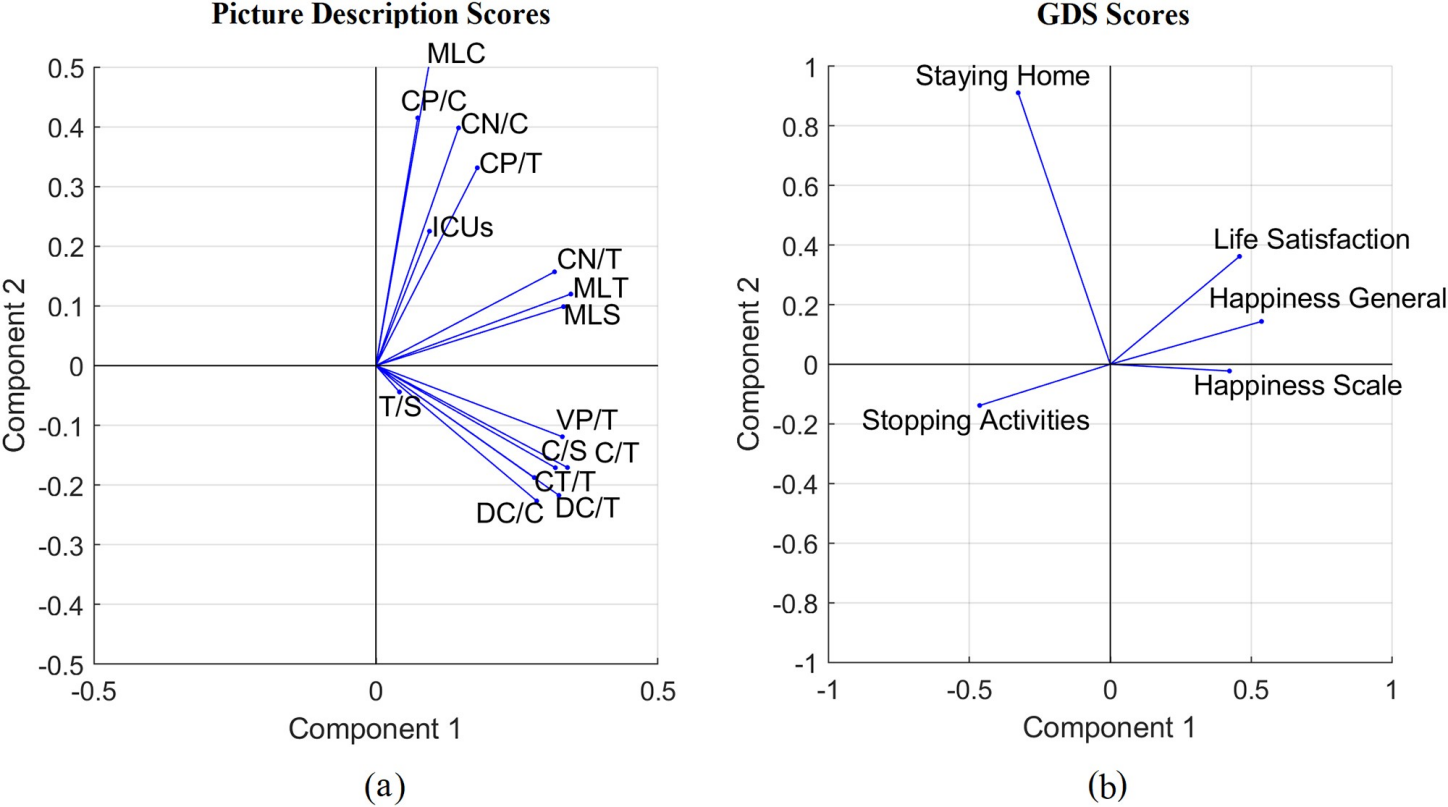
PCA analysis of different scores (a) Picture description, (b) GDS tasks.

Tasks such as GDS and picture description are scored based on different scoring metrics, which we can combine using principal components analysis. The picture description task includes features of both information content and language complexity. In Fig 13, the direction and length of the vectors indicate how each scoring metric contributes to the two principal components. For example, from Fig 13(a), the Dependent clause ratio (DC/C) and Dependent clauses per T-unit (DC/T), which reflect the amount of subordination, are approximately orthogonal to the Coordinate phrases per clause (CP/C) and Coordinate phrases per T-unit (CP/T), which reflect the amount of coordination. They are also approximately orthogonal to the number of ICUs. This suggests that DC/C and DC/T measure a very different aspect of the task compared to the CP/C and CP/T metrics. Similarly, from Fig 13(b) regarding GDS, the question about staying at home is approximately orthogonal to the other four questions, which are positively associated with happiness.

### Unsupervised analysis of data

**Fig 14:**
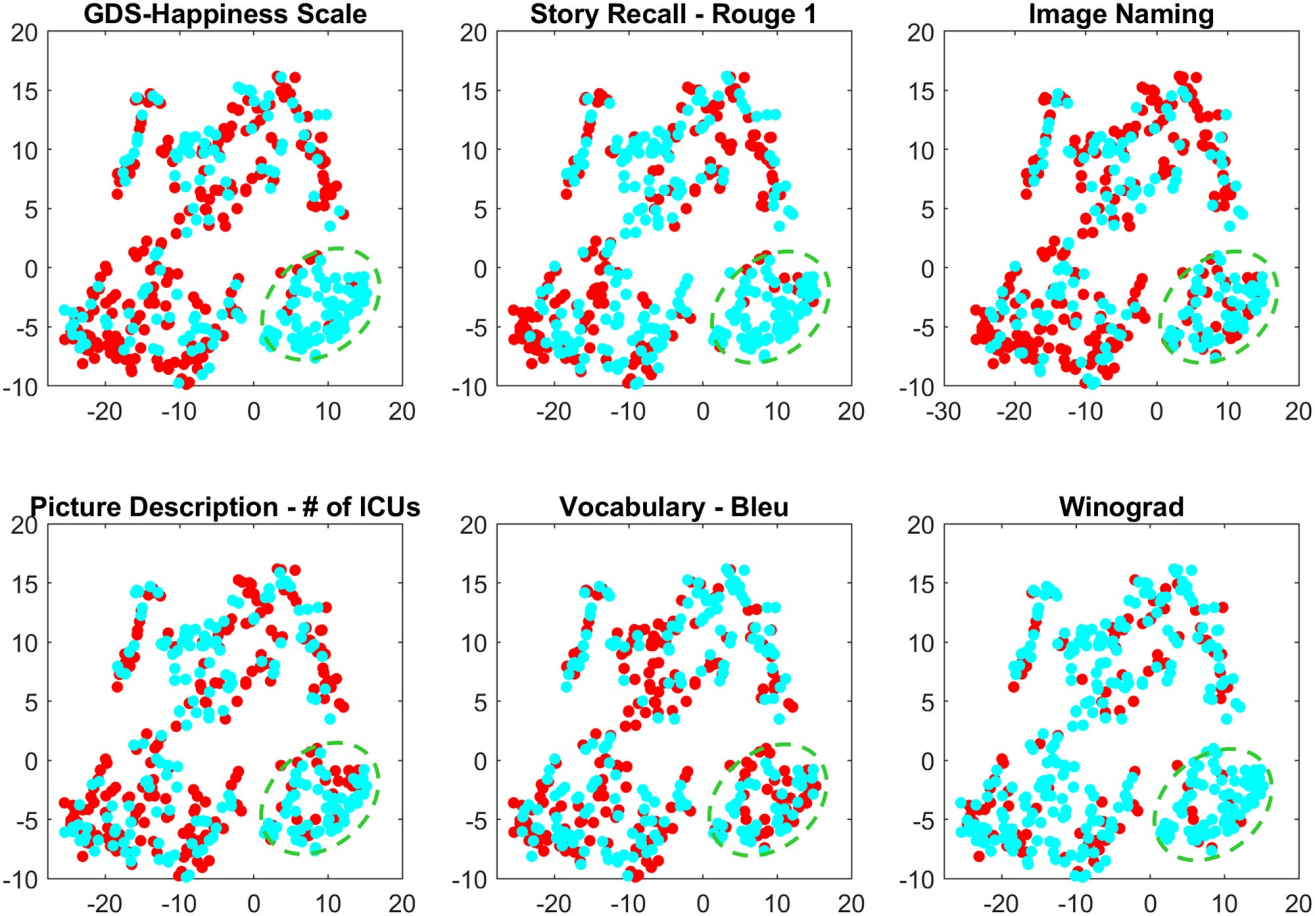
t-SNE reveals a cluster with high GDS happiness score. The cluster also shows high Rouge 1 scores. Scores higher (lower) than a threshold are in blue (red).

In order to further evaluate the generalizability of tasks involving spontaneous speech production, we look for the homogeneity across picture description and story recall tasks. We use t-SNE [76] to visualize *features* across these tasks. This analysis reveals a cluster, indicated with green ellipsoids across Fig 14. We further investigate characteristics of the cluster with respect to different *scores*. We colour the samples by comparing their score against a threshold, to highlight the homogeneity of the cluster with respect to that score. From Fig 14, it can be seen that the cluster is associated with high *GDS-Happiness scale* and high *story recall (Rouge-1)* score.

### Data augmentation for the assessment of Alzheimer’s disease

In this experiment, we combine our normative data with DementiaBank (DB), described above. We adopt the approach used by Vasquez-Correa *et al* [77] for multi-view representation learning via canonical correlation analysis (CCA) to improve the classification of dementia from healthy controls.

CCA computes a projection matrix for every view such that, in the shared space, the correlation between the projected samples from different views are maximized. We construct two views using the Talk2Me database. We consider features from the picture description task as the first view and the concatenation of features from the remaining tasks as the second view. Applying CCA on the Talk2Me database provides two projection matrices corresponding to the two views. We then project the DementiaBank data to the shared space using the projection matrix corresponding to the picture description task. We apply feature selection on the original features of DementiaBank and the selected features are concatenated with the CCA embeddings. Classification is done using an SVM with a radial-basis function kernel. Table 3 shows that, across five feature selection methods, the normative Talk2me data improves overall accuracy; however, an ANOVA test reveals no significant difference. We would encourage exploring additional tools in domain adaptation to handle domain shift and leverage complementary information could be a direction for future research. Moreover, the Talk2Me dataset may be more effective in application domains where participants have demographics more similar to Talk2Me. In the context of AD, it is important to recruit older adults. We will discuss this in the next section.

**Table 3:** Average accuracies (micro- and macro-) of the classification of dementia with and without CCA features. Columns refer to different feature selection methods.

	FMS [78]	Elasticnet [79]	KPLS [80]	Logo [81]	MetaDist [82]	No Feature Selection
Without CCA (micro)	76.3	74.8	76.7	77.6	73.7	60.7
With CCA (micro)	78.2	75.8	77.8	78.3	74.5	60.5
Without CCA (macro)	77.2	75.6	77.4	77.6	74.0	69.1
With CCA (macro)	79.0	76.4	78.6	78.2	74.9	68.6

Following [77], the number of embeddings is set to 20. We also examined a wide range of values for the number of embeddings (i.e., 10, 20, 30 and 40) and also different combinations of tasks to increase the number of views, but no significant difference was observed. We use 10-fold cross-validation in all cases. In addition, hyper-parameters are selected through an internal 10-fold cross-validation where the RBF kernel width is selected among [0.01, 1, 10, 100] and the error penalty parameter is set to 1. The number of selected features is selected among 50 to 300 in steps of 50. These settings are determined empirically.

## Limitations

We aim to design a platform that can be generalized to various populations, conditions, and tasks; in fact, we have recently applied it to a project involving language delays in elementary school children. However, a limitation of the existing data snapshot is that the age range is skewed towards young adults, and the majority of users have at least an undergraduate degree. For our work in specific demographics, e.g., in the detection of Alzheimer’s disease, it will be important to recruit more data from a wider range of people. Some potential barriers to recruitment include: older adults not knowing about the study, not being able to access the website, not wanting to put their personal information online, or not understanding the interface. These concerns may dissipate over time, as a growing proportion of older adults are using computers and accessing the Internet. For instance, Statistics Canada reports that Internet use among 65- to 74-year-olds rose from 65% to 81% in the period between 2013 and 2016, and from 35% to 50% in those aged 75 and older [83]. We intend to increase recruitment of this population through promoting the study on forums and mailing lists for older adults, and in retirement homes, assisted living facilities, and day programs.

Another limitation is the lack of control over recording conditions and environmental noise, which can present a challenge for audio processing. However, this is a consequence of collecting data with a set of microphones and recording conditions representative of the intended use. To be of practical use outside of controlled environments, analyses must be robust against changing channel conditions. In our reported analyses, we have previously attempted to mitigate such factors using spectral noise subtraction [84], and we have shown that software can reduce the effect of the channel in identifying differences in the voice [85]. Moreover, recent research suggests that “training on different noise environments and different microphones barely affects [speech recognition] performance, especially when several environments are present in the training data” [86].

The demographic and personal health information associated with the dataset are self-reported and have not been clinically validated. This can also be a limitation due to the potential for deliberate participant misrepresentation [87]. However, the cost and complexity associated with obtaining individual clinical assessments are not compatible with our goals of large-scale data collection and repeated, on-going participation.

## Conclusion and future work

We have developed a public portal for ongoing *longitudinal* language data collection from a naturalistic population—there are very few barriers to inclusion. We are releasing the first public ‘snapshot’ of normative data, consisting of 1033 sessions from 196 healthy subjects, including raw data, computed transcripts, features, and scores. We are also releasing a new software package (https://github.com/SPOClab-ca/COVFEFE) that extracts a variety of lexical, syntactic, semantic, pragmatic, and acoustic features for generic speech and language analysis. To our knowledge, this is the most comprehensive publicly available software pipeline for extracting linguistic features. The data and tools enable a common dataset to benchmark models, extend existing data sets with more data, including longitudinal data, and more diverse demographics and tasks. To describe these data, we analyze relations between tasks, and provide normative scores. This enables baselines against which smaller clinical data sets can be compared in the future. The Talk2Me dataset may be used to augment smaller datasets, especially those with demographics similar to Talk2Me. Along these lines, we have started to take a multi-view approach based on canonical correlation analysis, trained on Talk2Me, to improve the accuracy of classification [88].

We are currently recruiting more older adults to use the Talk2Me interface through various means, such as in retirement homes, assisted living facilities, and day programs. Also, we are currently extending the Talk2Me data collection tool to include a telephone-based interface. The telephone-based version of Talk2Me relies on interactive-voice-response and uses the same tasks as in the web-based version, except for Stroop. Data collection for both the web- and telephone- based systems is ongoing, and we are focusing our efforts on populations of older adults with and without dementia and cognitive decline.

## Supporting information

S1 Fig Demographics survey used on the talk2me website. (PDF)
