# Correction: Talk2Me: Automated linguistic data collection for personal assessment

**DOI:** 10.1371/journal.pone.0216375

**Published:** 2019-04-30

**Authors:** The PLOS ONE Staff

An incorrect version of S1 Fig was published in error. Please see the correct S1 Fig below.

In addition, the following information is missing from the Data Availability statement: All recordings, transcripts, computed features, and computed scores are also publicly available from: https://osf.io/dey8g/.

The publisher apologizes for these errors.

## Supporting information

S1 Fig Demographics survey used on the talk2me website. (PDF)
