# Supplementary material for: Talk2Me: Automated linguistic data collection for personal assessment
Source: PLoS One. 2019 Mar 27;14(3):e0212342. doi: 10.1371/journal.pone.0212342 (PMC6436678; doi:10.1371/journal.pone.0212342)
Supplement: S1 Fig — (PDF) [file pone.0212342.s001.pdf]

National Research Council Canada | Conseil national de recherches Canada

Licence to Publish (Crown Copyright)

Manuscript number: PONE-D-18-25016R2

No changes are to be made to this licence without prior consent of NRC Legal Services.

To: Public Library Of Science (PLOS)
1160 Battery Street
Koshland Building East, Suite 225
San Francisco, CA 94111 USA
(contact name and address of publisher)

This refers to the manuscript entitled (hereinafter the "manuscript"):
Talk2Me: Automated linguistic data collection for personal assessment

written by the author(s):
Majid Komeili, Chloe Pou-Prom, Daniyal Liaqat, Kathleen C Fraser, Maria Yancheva,
Frank Rudzicz

to be published in:
PLOS One

1. Definitions

   Accepted Manuscript:
   The version of a manuscript that has been accepted for publication following peer review. Content and layout follow publisher's submission requirements.

   Version of Record:
   A fixed version of an Accepted Manuscript that has been made available by a publisher. This includes any "early release" article that is formally identified as being published even before the compilation of a volume issue and assignment of associated metadata, as long as it is citable via some permanent identifier(s). This does not include any "early release" article that has not yet been "fixed" by processes that are still to be applied, such as copy-editing, proof corrections, layout, and typesetting.

2. The authors (except those listed in this paragraph) contributed to the manuscript on behalf of the National Research Council of Canada ("NRC"), thereby establishing a copyright belonging to the Crown in Right of Canada, that is, to the Government of Canada. NRC is not able to deal with the rights relating to the contribution of any co-authors listed below, and you should seek their permission separately [*may say* none].

   Majid Komeili (Carleton University); Chole Pou-Prom (LiKa Shing Knowledge Institute, Saint Michaels' Hospital); Daniyal Liaqat (Vector Institute & U of Toronto); Maria Yancheva (WinterLights Labs Inc); Frank Rudzicz (LiKa Shing Knowledge Institute, Saint Michael's Hospital, Vector Institute, University of Toronto and Surgical Safety Technologies)
   (list author names as well as their respective organization, email address and phone number, if available)

3. With respect to the Crown's copyright, NRC grants you permission to publish the Version of Record in the publication named above, on the condition that clear attribution is given to the authors and the National Research Council of Canada. After that publication, you may re-publish the Version of Record in any form or medium, with the same condition about attribution.

4. You are hereby [*specify* hereby *or* not] granted the right to license others to copy or publish the Version of Record. (If this right is granted, it is subject to the same condition about attribution).

5. NRC, for itself and those authors not listed in section 2, warrants that:
   a. The manuscript is the original work of the named author(s).
   b. A Version of Record has not been published elsewhere and will not be permitted to be published elsewhere, if you accept the manuscript for publication.
   c. The manuscript contains no infringing, libellous or other unlawful statements.

6. NRC retains the following rights, provided that when reproducing the manuscript or extracts from it, the publisher is acknowledged:
   a. To post a copy of the Accepted Manuscript on the NRC's website, the author(s)' funding body's designated archive and any other open access digital repository, subject to the embargo period specified herewith: [*specify* none, 3 months, 6 months *or* 12 months], none. For the purposes of clarity, where the embargo period is left blank, NRC will assume that a copy of the Accepted Manuscript may be posted immediately upon the date of acceptance of the manuscript for publication. A copy of the Version of Record will only be posted if so directed by the publisher. For this article, the publisher's preference is that we replace [*specify:* not replace *or* replace] the Accepted Manuscript with the Version of Record once the Version of Record becomes available.
   b. To reproduce the Accepted Manuscript for non-commercial purposes including other formats and other forms of expression.
   c. To reproduce the Accepted Manuscript for the purpose of education or research by the Government of Canada and to permit other institutions with which the author(s) is affiliated to reproduce the Accepted Manuscript for the purpose of education or research.
   d. To reuse figures, tables or an abstract created by the author(s).
   e. To authorize others to make any non-commercial use of the Accepted Manuscript so long as the author(s) receives credit as author(s) and the publication in which the Version of Record has been published is linked to the source of publication of the Version of Record (link to the publisher's or journal site).

7. It is believed that this authorization will provide you with all the scope of authority you require from the Government of Canada, but it does NOT transfer the copyright to you.

Signed at Ottawa, ON (City and Province/State) on 11-02-19 (Date)

NATIONAL RESEARCH COUNCIL OF CANADA

Per: [signature] (Director General)

John Shannon, Director General (acting), NRC-DT (Research centre)
