# Supplementary material for: Correction: Talk2Me: Automated linguistic data collection for personal assessment
Source: PLoS One. 2019 Apr 30;14(4):e0216375. doi: 10.1371/journal.pone.0216375 (PMC6490913; doi:10.1371/journal.pone.0216375)
Supplement: S1 Fig — (PDF) [file pone.0216375.s001.pdf]

Demographic Data

Please provide the following anonymous demographic data, which will aid the analysis of your results.

1. **Gender:**
   - ◯ Female
   - ◯ Male
   - ◯ Other : ________

2. **Date of birth (YYYY-MM-DD):**

   YYYY-MM-DD

3. **People in Canada come from many racial or cultural groups. You may belong to more than one group on the following list. Are you:**
   - ☐ White
   - ☐ Chinese
   - ☐ South Asian
   - ☐ Black
   - ☐ Filipino
   - ☐ Latin American
   - ☐ Southeast Asian
   - ☐ Arab
   - ☐ West Asian
   - ☐ Japanese
   - ☐ Korean
   - ☐ Aboriginal
   - ☐ Other

4. **Select all languages you can communicate in (speak, understand, read, and write), along with the level of fluency for each:**

Language	Fluency
☐ English	◯ Native<br>◯ Fluent<br>◯ Intermediate<br>◯ Beginner

Language	Fluency
☐ French	◯ Native<br>◯ Fluent<br>◯ Intermediate<br>◯ Beginner
Select another language...	◯ Native<br>◯ Fluent<br>◯ Intermediate<br>◯ Beginner

Add Another Language

5. **Education level (highest completed, or currently being completed)**

◯ University - Doctoral Degree
◯ University - Master's Degree
◯ University - Undergraduate Degree
◯ College
◯ High School
◯ Elementary School
◯ None

6. **Have you ever been diagnosed with dementia? Please select any type of dementia with which you've been diagnosed. If you've never been diagnosed, please leave this section blank:**

☐ Alzheimer's disease (AD)
☐ Vascular dementia (VD)
☐ Mild cognitive impairment (MCI)
☐ Dementia with Lewy Bodies (DLB)
☐ Frontotemporal dementia (FTD)
☐ Mixed dementia
☐ Parkinson's disease (PD)
☐ Other : ______

7. **Do you currently take any medications for dementia?**

◯ Yes
◯ No

8. **Have you been a regular smoker (of tobacco) within the last 3 years?**

◯ Yes

○ No

9. **Country you were born in:**

Select country...

10. **Country you currently reside in:**

Select country...

Submit

© University of Toronto 2018
